# St. Mark's Hospital

**Published:** 1914-08-15

**Authors:** 


### THE EDITOR'S LETTER-BOX.

### St. Mark's Hospital.

*To the Editor of* The Hospital.

Sir,—It is all very well, but Mr. Mummery does not state that Mr. Coope is a nephew of Mr. Sowden and has had the place kept warm for him for some time.

The advertisement was not given ordinary prominence.—Yours faithfully,

Another Hospital Officer.

### Poor-Law Infirmaries and the War.

*To the Editor of* The Hospital.

